# 879 Evaluating the Safety and Effectiveness of VTE Prophylaxis in Burn Patients: A Retrospective Study

**DOI:** 10.1093/jbcr/iraf019.410

**Published:** 2025-04-01

**Authors:** Katie Ronis, Jacob Denkins, Lindsay Desantis, Laurin Proctor, Samantha Allbritton, Brett Bird, Lily Daniali, Ryan Endress, Wojciech Przylecki, Benson Pulikkottil

**Affiliations:** HCA HealthONE Swedish; HCA HealthONE Swedish; HCA HealthONE Swedish; HCA HealthONE Swedish; HCA HealthONE Swedish; HCA HealthONE Swedish; HCA HealthONE Swedish; HCA HealthONE Swedish; HCA HealthONE Swedish; HCA HealthONE Swedish

## Abstract

**Introduction:**

Burn patients are at a heightened risk of venous thromboembolism (VTE). This study aimed to evaluate the impact of a standardized VTE prophylaxis protocol on VTE incidence and major bleeding rates in burn patients.

**Methods:**

A retrospective observational study was conducted, including adult burn patients admitted to a burn center over a 28-month period. The protocol mandated enoxaparin 40 mg twice daily for patients with ≥ 20% total body surface area (TBSA) burns weighing at least 40kg, or a BMI ≥ 40. Dose adjustments were calculated based on anti-Xa levels. The primary outcome was the comparison of VTE incidence before and after protocol implementation. The primary safety endpoint was major bleeding. Secondary outcomes included subgroup analysis by TBSA, VTE prophylaxis type, and protocol eligibility.

**Results:**

A total of 263 patients were included (pre-protocol: 118, post-protocol: 145). The overall VTE incidence was 2.5% pre-protocol and 4.1% post-protocol (P=0.521). Major bleeding occurred in 3.4% of pre-protocol and 2.8% of post-protocol patients (P=1.0). Subgroup analyses based on TBSA, VTE prophylaxis type, and protocol eligibility did not reveal statistically significant differences in VTE incidence.

**Conclusions:**

Implementation of the VTE prophylaxis protocol did not result in a statistically significant reduction in VTE incidence compared to prior standard care, despite a trend towards increased dose consistency. Further research is warranted to optimize VTE prophylaxis strategies in this high-risk patient population. Ongoing efforts are focused on evaluating protocol adherence and minimizing interruptions in prophylaxis to potentially improve outcomes.

**Applicability of Research to Practice:**

The optimal VTE prophylaxis in burn patients remains unknown. Although enoxaparin 40 mg twice daily has emerged as the most widely recommended dose, it is unknown which burn patients should utilize this dose. This study evaluates the efficacy and safety of a novel VTE prophylaxis protocol for burn patients. Although this current study did not show a reduction in VTE events, it hopefully brings the burn population one step closer to finding the ideal VTE prophylaxis. Further stratification of the data could be strengthened by employing a larger cohort to investigate co-morbidities, the extent of immobilization, and the incidence of VTE associated with line placements.

**Funding for the Study:**

N/A

